# Cardiac toxicity in patients with lung cancer receiving thoracic radiotherapy and immunotherapy

**DOI:** 10.3389/fonc.2022.1025455

**Published:** 2023-01-09

**Authors:** Christine Son, Melissa Y. Y. Moey, Paul R. Walker, Abdul R. Naqash, Matthew Sean Peach, Andrew W. Ju

**Affiliations:** ^1^ Brody School of Medicine at East Carolina University, Greenville, NC, United States; ^2^ Department of Cardiovascular Sciences at Vidant Medical Center/East Carolina University, Greenville, NC, United States; ^3^ Department of Hematology and Oncology at East Carolina University, Greenville, NC, United States; ^4^ Medical Oncology/TSET Phase 1 Program OU Health Stephenson Cancer Center at the University of Oklahoma, Oklahoma City, OK, United States; ^5^ Department of Radiation and Oncology at East Carolina University, Greenville, NC, United States

**Keywords:** immune checkpoint inhibitors (ICI), immunotherapy, radiotherapy, cardiotoxicity, cardiac radiation dose

## Abstract

**Background:**

Immune checkpoint inhibitors (ICIs) are used to treat locally-advanced and metastatic lung cancer, which can lead to severe immunogenic-related cardiotoxicities. We assessed the risk of cardiotoxicity in ICI-treated lung cancer patients with or without cardiac radiation from thoracic radiotherapy.

**Methods:**

Retrospective data was collected on Stage III-IV lung cancer patients who received ICIs between 2015 and 2018. All cardiotoxicities associated with ICI were assessed in correlation with the timing of radiotherapy (RT) in relation to ICI, and the mean RT heart dose. The rate of cardiac events in relation to RT timing and heart dose was compared using multiple logistic regression including the Framingham risk score and steroid use prior to ICI therapy.

**Results:**

Of 194 ICI-treated patients evaluated, 55.2% (n=107/194) patients had received thoracic RT at a median dose of 60.4 Gy (range, 15-75). Cardiotoxicities such as non-ST elevated myocardial infarction and new onset supraventricular tachycardias were observed in 13 (12.2%) of those who had thoracic RT versus 9 (10.3%) who did not (p=0.87). 38 patients who received RT concurrently with ICI did not develop any cardiotoxicity whereas 14.1% (n=22/156) of those who did not receive concurrent RT developed cardiotoxicities (univariate, p=0.030; multivariate, p=0.055). There were no significant differences in the mean heart RT dose, Framingham risk score, and steroid treatment between patients that received concurrent RT with ICI versus non-concurrent RT/ICI.

**Conclusion:**

ICI-related cardiotoxicities were not significantly associated with patients who received concurrent thoracic radiotherapy in this retrospective review. Further validation of prospective studies is needed to confirm these results.

## Introduction

Immune checkpoint inhibitors (ICIs) and chimeric antigen receptor T-cell therapy have been successful in treating a wide variety of cancers including melanoma, lymphoma, and lung cancer (1). ICIs amplify T-cell-mediated immune responses against cancer cells by blocking intrinsic immune down-regulators such as anti-cytotoxic T-lymphocyte associated protein 4 (CTLA-4), programmed cell death protein-1 (PD-1), and programmed cell death ligand-1b (PD-L1) (1). While surgical resection remains the standard of care for early-stage non-small-cell lung cancer (NSCLC), chemoradiation has been a mainstay of treatment for locally advanced NSCLC. Consolidation immunotherapy has emerged as the new standard with improved survival (2) in this subset of patients after conventional chemoradiation, with the regimens including CTLA-4, PD-1, and PD-L1 checkpoint inhibitors.

Radiation in high doses may potentiate an immune response called the abscopal effect, in which local treatment of radiation therapy (RT) induces a systemic response that could target distant sites of the disease (3). Conversely, radiation may also induce immunosuppression through the T-cell inhibition (4). Concurrent chemoradiotherapy in patients with metastatic disease may reduce the time to recurrence and may improve overall survival, however, with a potentially increased risk for immune-related adverse events (irAEs) (3, 5). One of the rare irAEs in combination with RT is ICI-related myocarditis, a potential therapy-limiting form of cardiotoxicity with a high rate of associated significant morbidity and mortality (1).

Du et al. demonstrated strong preclinical evidence that radiation-induced cardiotoxicity is modulated by the PD-1 axis by delivering cardiac irradiation in a mouse model concurrently with PD-1 blockade (6). Radiation was delivered in a single dose of 20 Gy or in fractionated cardiac irradiation of 30 Gy delivered over five fractions. Acute mortality of 30% within 2 weeks after cardiac irradiation plus anti–PD-1 antibody compared with 0% from cardiac irradiation plus immunoglobulin G (p=0.023) was observed, which was also associated with decreased cardiac output, increased lymphocytic infiltration, and fibrosis (6). There is a paucity of data reporting cardiotoxicity in patients receiving both RT and immunotherapy, especially in the real-world setting. The objective of this study was to assess if the interaction between cardiac heart dose from thoracic RT and immunotherapy in lung cancer patients was correlated with different clinical outcomes.

## Methods

Patients with a diagnosis of NSCLC and small-cell lung cancer (SCLC) who received ICIs in the past 3 years between February 2015 and February 2018 were identified from the Tumor Registry at Vidant Medical Center/East Carolina University. Patients were included in the study if they were ≥ 18 years old, completed at least 1 infusion of ICI therapy, and received treatment at our institution. RT was delivered between cycles of immunotherapy for patients who received RT concurrently with ICI. Concurrent RT with ICI has been delivered within our institution especially if the patient required palliative therapy. Usually, acute radiation side effects can last up to 12 weeks after radiation (7), and the timing of cardiotoxicity typically occurs early after initiation of ICI with a median time of 2 months with the majority of the cases occurring within 3 months (8). Therefore, we looked to see when acute toxicities are most likely to be present by comparing the variability in the overlaps between radiation therapy and immunotherapy.

Demographic data including age, sex, body mass index, and ethnicity were collected. Information regarding the patient’s lung cancer type, prior and concomitant chemotherapy, ICI therapy, medical history, cardiac medications prior to initiation of therapy, steroid use prior to initiation of therapy, and cardiac radiation dose were retrospectively reviewed. Thoracic radiation parameters collected included prescription dose, dose per fraction, and mean heart dose. Dosimetric parameters V5, V25, and V30 were also collected.

ICI-related cardiotoxicities (iRC) (+) patients were defined as 1) non-ST elevated myocardial infarction (NSTEMI) in the setting of acute cardiac syndrome symptoms, 2) new onset supraventricular tachycardias (SVT) including atrial fibrillation (AF), 3) myocarditis or 4) pericardial disorders (acute pericarditis and/or non-malignant pericardial effusion) in the absence of sepsis, electrolyte disorders or other confounding medical problems. A clinical diagnosis of myocarditis included 2 or more clinical presentations of: 1) new-onset (0 days up to 3 months) or subacute/chronic (>3 months of worsening dyspnea at rest/exertion and/or fatigue with left and/or right heart failure signs and/or imaging findings of new right and/or left ventricular dysfunction, or elevations of natriuretic peptides or troponin 2) cardiac death or aborted cardiac death 3) new I to III degree atrioventricular block or bundle branch block, sinus arrest, ventricular tachycardia or fibrillation and asystole, or 4) tissue characterization by cardiac magnetic resonance imaging (MRI) showing edema or late gadolinium enhancement. iRC (-) patients were those who did not experience a cardiac-related irAEs and were further subcategorized into patients who did not experience any irAEs, experienced non-cardiac irAEs, or who had disease progression (could not complete 4 cycles of ICI due to tumor progression).

Cardiotoxicity that could be attributed to ICI was determined by cardiologists and medical oncologists and graded based on the common terminology for clinical adverse events (CTCAE) (**Table 1**). The cohort used in the analysis is a subset of patients described in a prior report on cardiotoxicity and ICI that did not analyze the interaction with radiotherapy. The methodology of grading toxicity is described in this publication (9). The Framingham risk score for predicting 10-year coronary heart disease risk was calculated according to the modified model in 2008 (10). Chi-square was used for data with binary inputs. Student’s t-test was used for continuous data including baseline age and BMI. Mann-Whitney U-test was used for non-parametric data including mean heart dose and Framingham Risk Score. Multiple regression was used for multivariate analysis. All statistical analyses were performed using MedCalc version 12.6.0 (MedCalc Software, Ostend, Belgium).

**Table 1:** CTCAE toxicity grading scale for iRC.

CTCAE Term	Grade 1	Grade 2	Grade 3	Grade 4	Grade 5
Myocardial infarction	–	Asymptomatic and cardiac enzymes minimally abnormal and no evidence of ischemic ECG changes	Severe symptoms; cardiac enzymes abnormal; hemodynamically stable; ECG changes consistent with infarction	Life-threatening consequences; hemodynamically unstable	Death
Supraventricular tachycardia	Asymptomatic, intervention not indicated	Non-urgent medical intervention indicated	Symptomatic, urgent intervention indicated	Life-threatening consequences	Death
Atrial fibrillation	Asymptomatic, intervention not indicated	Non-urgent medical intervention indicated	Symptomatic, urgent intervention indicated; device (e.g., pacemaker); ablation; new onset	Life-threatening consequences; embolus requiring urgent intervention	Death
Myocarditis	–	Symptoms with moderate activity or exertion	Severe with symptoms at rest or with minimal activity or exertion; intervention indicated; new onset of symptoms	Life-threatening consequences; urgent intervention indicated (e.g., continuous IV therapy or mechanical hemodynamic support)	Death
Pericarditis	Asymptomatic, ECG or physical findings (e.g., rub) consistent with pericarditis	Symptomatic pericarditis (e.g., chest pain)	Pericarditis with physiologic consequences (e.g., pericardial constriction)	Life-threatening consequences; urgent intervention indicated	Death
Pericardial effusion	–	Asymptomatic effusion size small to moderate	Effusion with physiologic consequences	Life-threatening consequences; urgent intervention indicated	Death

Common terminology criteria for adverse events. Version 5.0 March 2018. Available at: (3). Accessed November 11, 2019).

## Results

### Baseline patient characteristics and radiation therapy

Among 194 patients reviewed retrospectively, 176 patients had NSCLC and 18 patients had SCLC. Patients had a median age of 64 (IQR of 36 to 88 years old), and were predominantly Caucasian (n=123, 63.4%) and male (n=112, 57.7%). Patients had either stage III (n=66, 34.02%) or stage IV (n=128, 65.98%) lung cancer. The median Framingham score for our patient population was a 22.34% risk of a cardiac event in 10 years. Some of the predominant cardiac comorbidities included diabetes in 29.38% of patients, coronary artery disease/coronary artery bypass graft in 17.01% of patients, hypertension in 62.89% of patients, hyperlipidemia in 30.41% of patients, and chronic obstructive pulmonary disease in 36.59% of patients. Medications included steroids, beta-blockers, calcium channel blockers, diuretics, renin-angiotensin-aldosterone-system inhibitors, and statins (**Table 2**).

**Table 2:** Baseline patient characteristics include demographics, cancer history, co-morbidities, cardiac medical history, and medications prior to initiation of therapy.

Total Number of Patients	194
Received thoracic RT	107 (55.15%)
Did not receive thoracic RT	87 (44.85%)
	
**Demographics, n (%)**	
Age (median [IQR]), years old	64 (36-88)
Male	112 (57.73%)
Female	82 (42.47%)
White	123 (63.40%)
Black	69 (35.57%)
Other	2 (1.03%)
	
**Lung Cancer History, n (%)**	
NSCLC	176 (90.72%)
SCLC	18 (9.28%)
	
**Lung Cancer Stage, n (%)**	
Stage III	66 (34.02%)
Stage IV	128 (65.98%)
	
**Baseline Cardiac History, n (%)**	
Diabetes	57 (29.38%)
Coronary artery disease/Coronary artery bypass graft	33 (17.01%)
Hypertension	122 (62.89%)
Hyperlipidemia	59 (30.41%)
Atrial fibrillation/Atrial flutter	18 (9.28%)
Aortic insufficiency	9 (4.64%)
Chronic obstructive pulmonary disease	71 (36.59%)
Congestive heart failure	18 (9.28%)
Peripheral arterial disease	5 (2.58%)
Hypothyroidism	14 (7.22%)
Cerebral vascular disease	22 (11.34%)
Chronic kidney disease	10 (5.15%)
Abdominal aortic aneurysm	8 (4.12%)
Deep vein thrombosis/Pulmonary embolism	16 (8.25%)
Obstructive sleep apnea	10 (5.15%)
	
**Baseline Cardiac Medications**	
Steroids	83 (42.78%)
Beta-blockers	67 (34.54%)
Calcium channel blockers	38 (19.59%)
Diuretics	56 (28.87%)
Renin-angiotensin-aldosterone-system inhibitors	45 (23.2%)
Statins	69 (35.57%)

Patients received different chemotherapy regimens with the majority receiving alkylating agents (n=135, 69.59%). Majority of the patients received nivolumab (n=135, 69.59%) while remaining patients received pembrolizumab (n=48, 24.74%) and atezolizumab (n=11, 5.67%). The median total number of ICI cycles patients received was 4, range of 1-27. In patients who developed cardiotoxicity, the median number of ICI cycles they received prior to the cardiotoxic event was 3.5 cycles, range of 1-8. The average period of monitoring for toxicity after the initiation of ICI was 5.6 months, counting after the date of the first infusion, and 22.2 months, counting after the last date of the thoracic RT. The average total prescription dose of thoracic RT was 60.4 Gray (Gy). The mean dose per fraction of thoracic RT was 1.8 Gy, and the range was 1.3-12 Gy. The average global heart dose was 9.53 Gy, range of 0-37.74 Gy. The average maximum point dose to the heart was 63.38 Gy. The mean heart dosimetric parameters were 36.85 (range 0-100) for V5, 15.22 (range 0-67) for V25, and 11.51 for V30 (range 0-55) (**Table 3**).

**Table 3:** Chemotherapy and ICI therapy stratification and radiation treatment parameters.

**Chemotherapy, n (%)**	
Anti-topoisomerase	80 (41.24%)
Anti-VEGF	13 (6.7%)
Alkylating agents	135 (69.59%)
Anti-metabolites	75 (38.66%)
Texanes	16 (8.25%)
	
**ICI Therapy, n (%)**	
Nivolumab	135 (69.59%)
Pembrolizumab	48 (24.74%)
Atezolizumab	11 (5.67%)
	
**Total Number of ICI cycles received** (Median, range)	4 (1-27)
**Number of ICI cycles received prior to cardiotoxic event**	3.5 (1-8)
	
**Average period of monitoring after ICI therapy**	
after the date of the thoracic RT end date	22.2 months
after the initiation of ICI	5.6 months
	
**Radiation dose (Gy)**	
Total prescription dose of thoracic RT (mean and range)	60.4 (15-75)
Dose per fraction of thoracic RT (median and range)	1.8 (1.3-12)
Mean heart dose (mean and range)	9.53 (0-37.74)
Heart V5 (median and range)	36.85 (0-100)
Heart V25 (median and range)	15.22 (0-67)
Heart V30 (median and range)	11.51 (0-55)
Max point dose to heart (median and range)	63.38 (0.22-73.4)

### Cardiotoxicity in patients receiving ICI and RT

**Figure 1:**
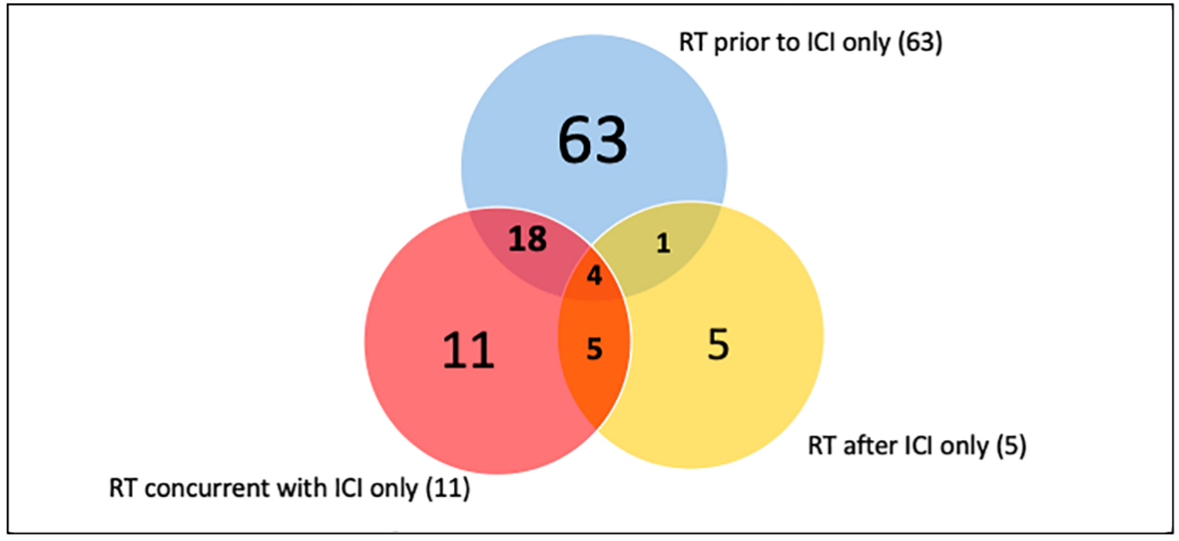
Thoracic RT and ICI treatment schedule. The number indicates a number of patients treated with thoracic RT in regards to the ICI schedule.

**Figure 2:**
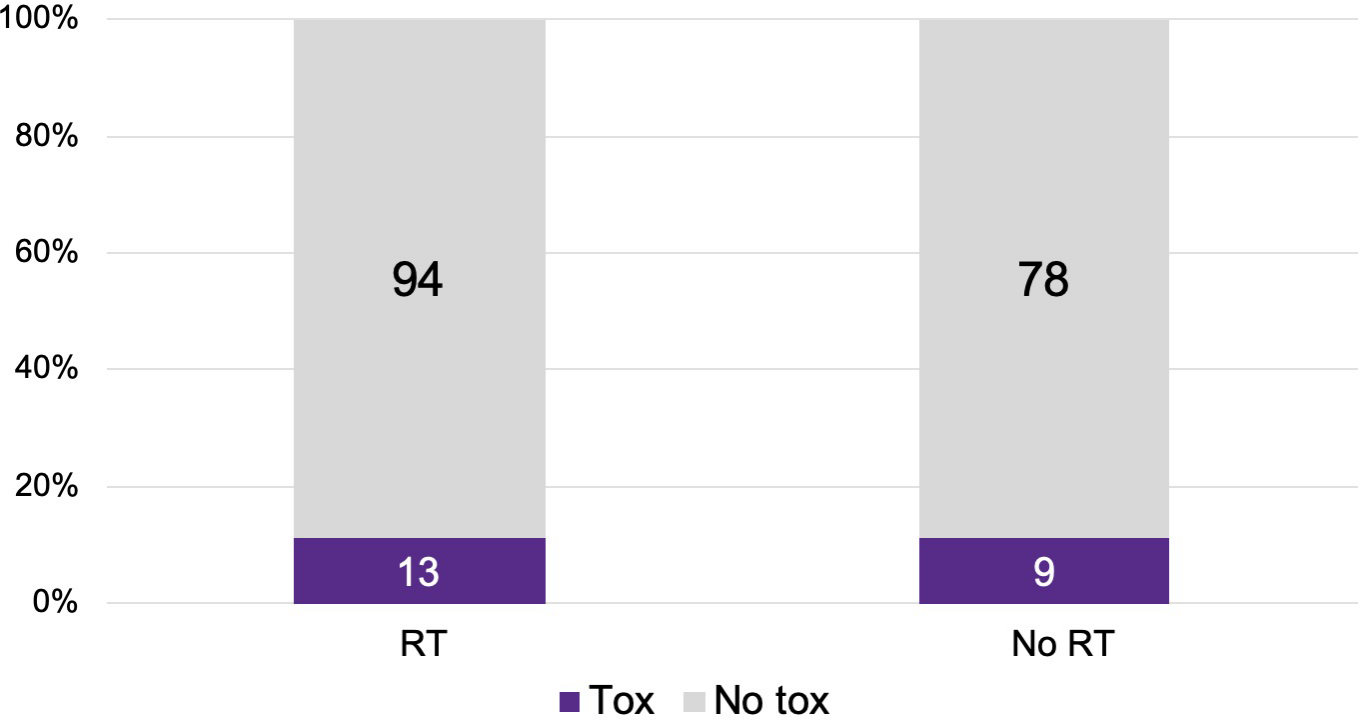
The plot of cardiotoxic events between the 107 patients who received thoracic RT versus 87 patients who did not receive thoracic RT. The numbers indicate patients who did (purple) or did not (gray) experience a cardiotoxic event. There was no significant difference in the percentage of patients with cardiotoxic events between the 2 groups (p=0.87).

**Figure 3:**
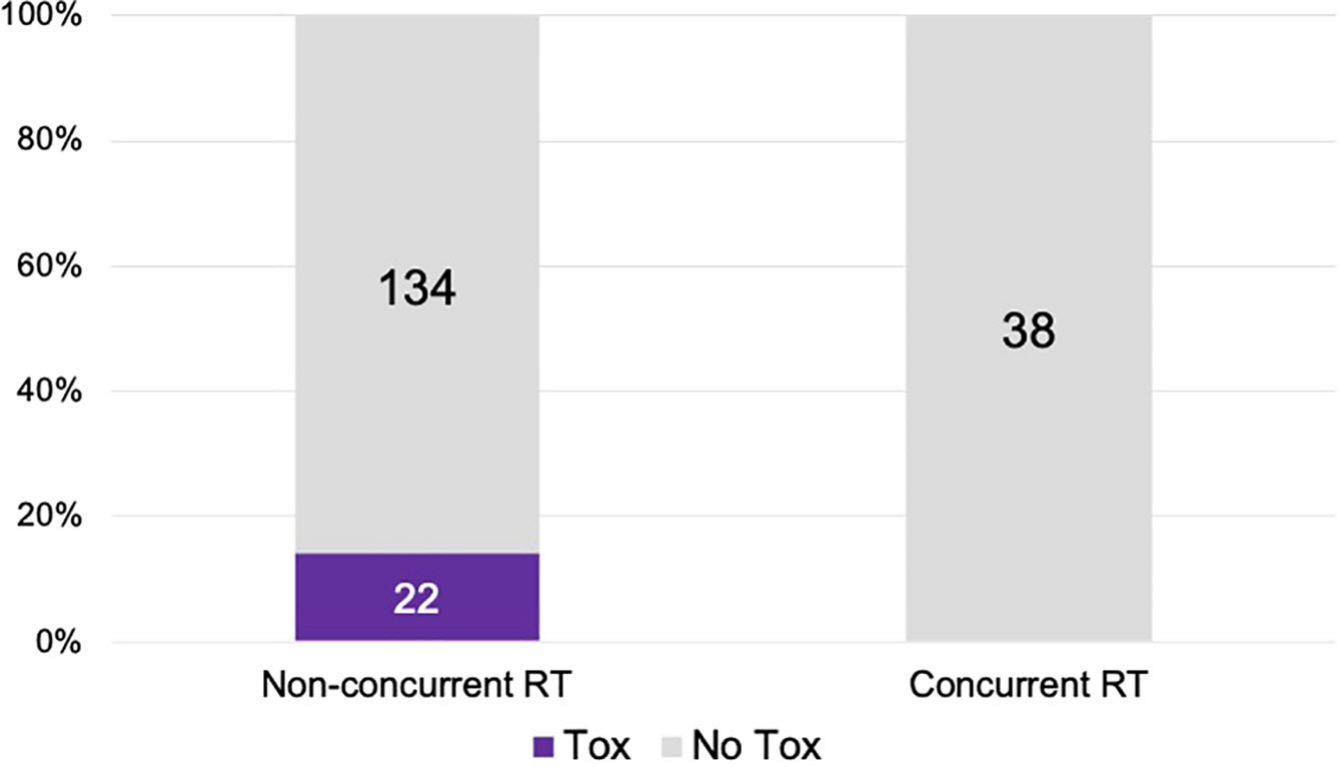
The plot of cardiotoxic events in patients who received ICI concurrent with thoracic RT versus ICI not concurrently with thoracic RT. Patients who received ICI non-concurrently with thoracic RT had higher rates of cardiotoxicity (p=0.030).

**Figure 4:**
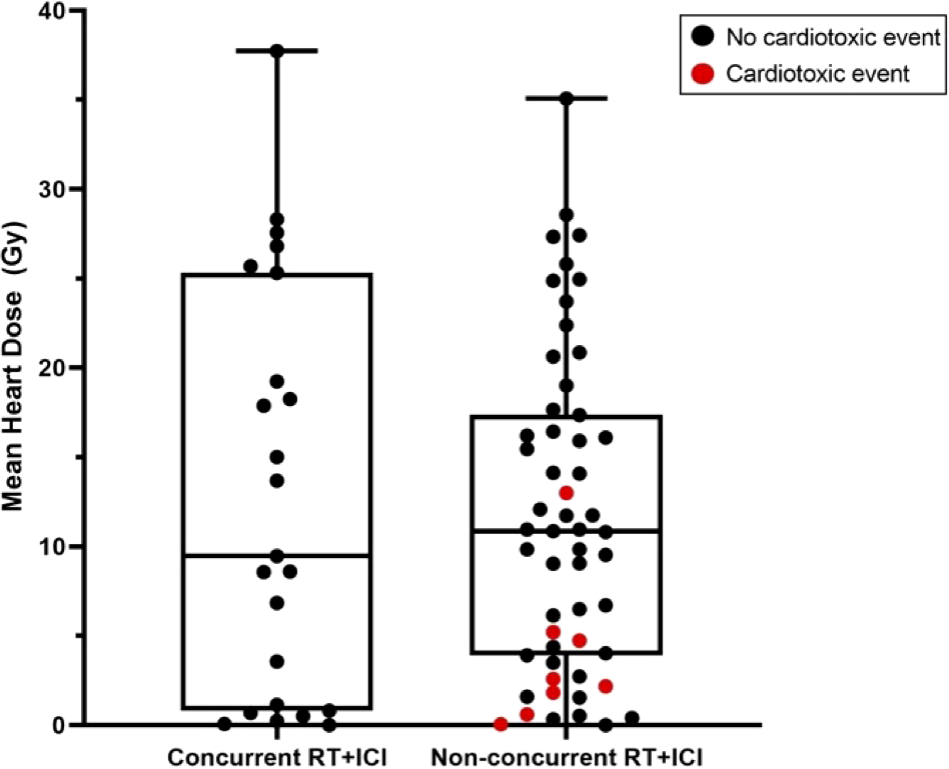
Box and whisker plots of mean heart dose of subjects who received radiotherapy concurrently with ICI (concurrent RT+ICI) versus not (non-concurrent RT+ICI). Each point represents a patient, plotted against the range, interquartile range, and median. There was no significant difference in mean heart dose between the groups (p=0.96).

**Figure 5:**
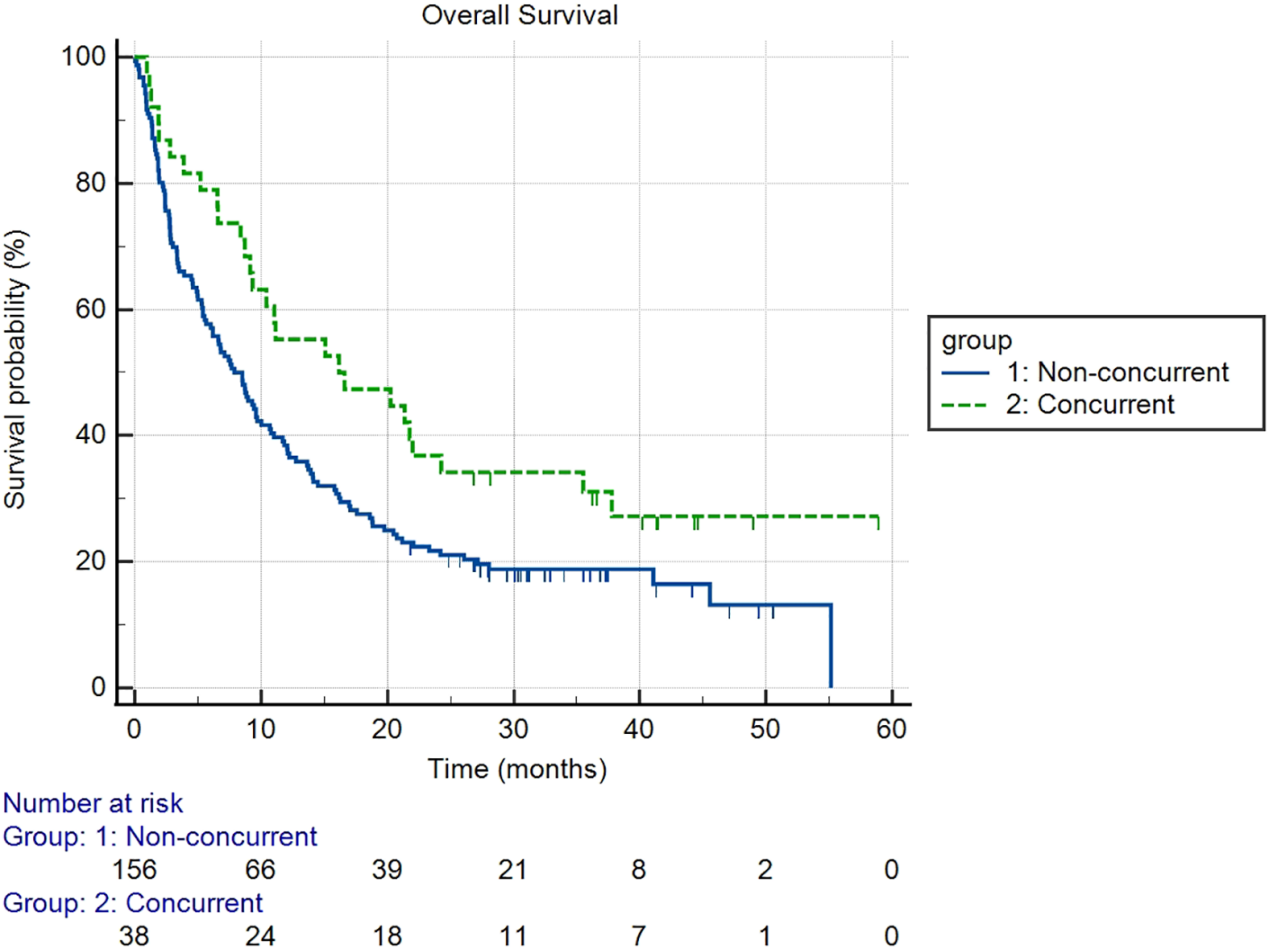
Kaplan-Meier survival analysis of the overall survival between the non-concurrent RT+ICI and concurrent RT+ICI. There was no significant difference in overall survival between the two groups (p=0.21).

There were 107 patients who received thoracic RT before, concurrently, or after ICI infusion, while 87 patients did not receive any thoracic RT (**Figure 1**). Among the 194 ICI-treated patients, 22 patients (11.3%) developed cardiotoxicity (**Figure 2**). **Table 4** describes the different type of cardiac toxic events that were described in these 22 patients. The two most common cardiac toxicities included myocarditis and cardiac arrhythmias such as SVT/AF. Among the patients who received thoracic RT, 38 (19.59%) patients received thoracic RT concurrently with ICI (**Figure 3**). **Table 5** describes the baseline patient characteristics between the cohorts of patients treated concurrently and non-concurrently. There was no statistical significance between the two groups regarding their age, gender, smoking status, coexisting cardiovascular disease including diabetes and the treatment for HTN, and prior known vascular disease. There was a statistical difference in their baseline BMI (p=0.008). Cardiotoxicity occurred in 13 patients (12.2%) out of 107 patients who received any thoracic RT versus 9 (10.3%) out of 87 patients who did not receive thoracic RT (p=0.87) (**Figure 2**). All 38 patients who received concurrent RT with ICI did not develop cardiotoxicity compared to 22 out of 156 patients (14.1%) who did not receive concurrent RT (p=0.030) (**Figure 3**). There was no significant difference in mean heart dose between the concurrent RT/ICI group versus the non-concurrent RT/ICI group (p=0.96) (**Figure 4**). There was no significant difference in the Kaplan-Meier survival analysis of the overall survival between the non-concurrent RT+ICI group and concurrent RT+ICI group (p=0.21) (**Figure 5**).

**Table 4:** Cardiac toxic events stratification in 22 patients.

Cardiac toxic event, n (%)	
NSTEMI/STEMI	4 (18.18%)
SVT/AF	9 (40.91%)
Myocarditis	13 (59.1%)
Pericardial effusion	3 (13.64%)
CHF exacerbation	1 (4.55%)

**Table 5:** Baseline patient characteristic comparison between the non-concurrent group and the concurrent group.

Comparison	Non-concurrent group (n=156)	Concurrent group (n=38)	*p*-value
Age (median, range)	65 (36-88)	61.5 (36-80)	0.59
Male (n, %)	91 (58.33%)	21 (55.26%)	0.73
BMI (median, range)	25.33 (13.25-85.73)	26.21 (17.44-39.07)	**0.008**
Smoking status (n, %)	56 (35.89%)	12 (31.58%)	0.61
Diabetes (n, %)	44 (28.21%)	13 (34.21%)	0.46
Under HTN treatment (n,%)	72 (46.15%)	21 (55.26%)	0.31
Known vascular disease (n, %)	50 (32.05%)	8 (21.05%)	0.18

There was no significant difference between the non-concurrent group and the concurrent group regarding the baseline patient characteristics, except for their baseline BMI (p=0.008). The p-values were calculated using the Chi-square for dichotomous values and using the Student’s t-test for continuous variables. For the Framingham risk score analysis, please refer to **Table 6**.

Two different cohorts were used for univariable analysis between patients who received ICI concurrently and non-concurrently with thoracic RT. Cohort 1 included patients who received some form of RT (n=107) while cohort 2 included all the patients, those with or without RT (n=194). In Cohort 1, there was no significant difference in mean heart RT dose (p=0.96), Framingham risk score (p=0.56), and prior steroid treatment (p=0.59) between patients that received concurrent RT with ICI versus non-concurrent RT/ICI. However, patients who received ICI non-concurrently with thoracic RT had significantly increased cardiotoxicity (p=0.044) (**Table 7**). In Cohort 2, there were no significant differences in mean heart RT dose (p=0.68), Framingham risk score (p=0.78), and prior steroid treatment (p=0.72) between patients that received concurrent RT with ICI versus non-concurrent RT/ICI. However, patients who received ICI non-concurrently with thoracic RT also had significantly increased cardiotoxicity (p=0.030) in this cohort (**Table 6**). In a multiple logistic regression model, steroid treatment was independently predictive of cardiotoxicity (p<0.001), and the lack of concurrent RT/ICI approached significance (p=0.055).

**Table 6:** Univariate analysis between patients who received ICI concurrently versus non-concurrently with thoracic RT.

Comparison	Non-concurrent group (n=156)	Concurrent group (n=38)	*p*-value	Method
Cardiotoxic event	22	0	**0.030**	Chi-square
Mean heart dose (Gy)	10.49	12.01	0.68	Mann-Whitney
Framingham score (median)	22.40%*	21.49%*	0.78	Mann-Whitney
Prior steroid use	65	14	0.72	Chi-square

Any grade of toxicity was included based on the CTCAE version 5.0. Cohort 2 includes all the patients included in the study, those with or without RT (n=194).

On multivariate analysis using multiple logistic regression in a backwards inclusion model, concurrent RT still approached significance (p=0.06) predicting reduced cardiotoxicity, while steroid use during ICI was significantly associated with increased cardiotoxicity (p<0.001). Mean heart dose and Framingham risk score were not retained as significant factors influencing major adverse cardiac events (MACE) rates.

*Framingham analysis excluded 76 patients with missing data for the non-concurrent group (48.72%) 15 patients with missing data for the concurrent group (39.47%). Bold values are statistically significant.

**Table 7:** Univariate analysis between patients who received ICI concurrently versus non-concurrently with thoracic RT.

Comparison	Non-concurrent group (n=78)	Concurrent group (n=29)	*p*-value	Method
Cardiotoxic event	13	0	**0.044**	Chi-square
Mean heart dose (Gy)	11.69	12.87	0.96	Mann-Whitney
Framingham score (median)	18.91%*	24.03%*	0.56	Mann-Whitney
Prior steroid use	33	9	0.59	Chi-square

Bold values are statistically significant. *Framingham analysis excluded 37 patients with missing data for the non-concurrent group (47.43%) 13 patients with missing data for the concurrent group (44.83%). Any grade of toxicity was included based on the CTCAE version 5.0. Cohort 1 includes patients who received some form of RT (n=107).

## Discussion

We assessed the relationship between cardiac radiation and risk of cardiotoxicity in NSCLC and SCLC patients treated with combined ICI and thoracic radiotherapy. Our results were not consistent with our initial hypothesis that concurrent treatment of RT with ICI worsens cardiotoxicity in lung cancer patients. In fact, the results showed that no MACEs occurred in patients who received thoracic radiotherapy concurrently with ICI. This suggests that thoracic radiation concurrently delivered with ICI does not necessarily increase the risk of cardiotoxicity. The fewer cardiac events in our cohort with thoracic radiotherapy ICI–treated patients were independent of the cardiac radiation dose, Framingham risk score, and prior steroid treatment. The baseline BMI was statistically different between the concurrent and non-concurrent groups but the higher BMI in the concurrent group, which would predict a higher risk of a cardiac event in that group, was not shown in our results.

The PACIFIC trial was the first phase III prospective randomized controlled trial to show an increase in progression-free survival (PFS) and overall survival (OS) using consolidation durvalumab following concurrent chemoradiation (2). The initial analysis showed a significantly improved PFS compared to the placebo (16.8 months compared to 5.6 months, p<0.001), which led to the FDA and EMA approval of duravalumab for use in the consolidative therapy (2). Upon the success of chemoradiation followed by immunotherapy in treating locally advanced NSCLC, many trials are investigating the incorporation of immunotherapy with concurrent chemoradiotherapy in the definitive management of locally advanced NSCLC. The preliminary safety and efficacy results of the PD-L1 inhibitor durvalumab in combination with conventional radiotherapy were assessed by number of small Phase I/II trials. Levy et al. recently showed that this combination was well tolerated in a small cohort of patients in their single center subset analysis in France (11). In a completed trial by Jabbour et al., concurrent PD-1 treatment with chemoradiotherapy was well tolerated with encouraging PFS of 69.7% at 12 months, however, with potentially an increased risk of pneumonitis using concurrent PD-1 checkpoint blockade (5). Therefore, much remains to be addressed about this combination, including the best sequences to follow when combining RT with ICIs and the best radiation dose for maximal efficacy (12, 13). Additionally, complicating the use of thoracic RT and ICI’s is that cardiac radiation has a dose dependent association with an increased risk of MACEs (14, 15).

The synergy of combining RT and immunotherapy to enhance clinical benefit through abscopal effects has been widely investigated in both pre-clinical and clinical settings (3). The local treatment of RT stimulates expression of MHC I, which primes T-cell response *via* release of tumor-specific antigens and immunomodulatory cytokines (16). The release of cytokines activates the systemic immune response against tumors even in the non-irradiated lesions, culminating into an abscopal effect. The addition of immunotherapy is thought to further enhance the abscopal effects by activating the immune system, which involves increase in lymphocytes. Immunotherapy such as immune checkpoint inhibitor (ICI) blocks the inhibition of T cells on antigen presenting cells, thereby unleashing T cells to react against tumor cells. ICIs are frequently administered intravenously, which allows for systemic activation of T cells, and when combined with RT-induced T cell priming effects, could potentiate antitumor immunity *via* abscopal effects, although this is not an effect that has been clearly demonstrated in human trials (17). However, radiation therapy-induced lymphopenia in patients treated with ICI may decrease the abscopal effects and leads to poor overall survival outcomes (18, 19). Chen et al. reported that patients with absolute lymphocyte count above median value despite RT had greater abscopal response and improved survival in response to ICI (20). In pancreatic cancer, Wild et al. (19) demonstrated that stereotactic body radiation therapy (SBRT), compared to conventional RT (CRT), delivers radiation dose in a smaller target volume with fewer fractions, suggesting lymphocytes in normal tissue could be preserved. Menon et al. also suggested patients who received low-dose radiation to metastatic lesions in combination with high-dose stereotactic ablative radiotherapy and ICI therapy may be capable of enhancing an immune response leading to abscopal effects (21). Our study did not distinguish between CRT and SBRT in association with the observed cardioprotective effects in ICI-treated lung cancer patient.

An underlying mechanism of immunotherapy-related cardiotoxicity is upregulation of CD8+T cells and CD68+ macrophages, involving hyperactivated cytotoxic T-cells (22). Although the mechanism is not completely understood, there is evidence that ICI increases T cells activities against common antigens shared by tumor and heart, leaving cardiac cells susceptible to cytotoxic T-cells (22). Therefore, RT may be effective in reversing such cardiotoxicity by decreasing lymphocyte count (22) as well as neutralizing immune activities by TGFβ induction as described by Vanpouille-Box et al. (4).

Most studies on combining RT and ICI therapy have focused on overall safety and efficacy. Implications specifically on cardiotoxicity with the combination of RT and ICI warrants further investigation on a molecular level. By better understanding the immunological mechanism of the combination in respect to cardiotoxicity may help formulate a safer treatment option for lung cancer patients who are good candidates for ICI therapy.

## Limitations

Given the retrospective study design, there may be under-reporting of cardiac events due to competing risk of patients’ metastatic, locally advanced disease diagnoses. The metastatic disease may interfere with patients’ attribution of cardiac symptoms as true cardiac symptoms, as most patients, especially if they received palliative treatments, may have symptoms that are attributed to their metastatic disease. It should be noted that this study was conducted with small sample size. The decrease in MACEs in patients receiving concurrent RT during ICI was based on univariate and multivariate (approaching significance) analyses. This may be due in part to insufficient data for a portion of the subjects leading to incomplete Framingham risk scoring, thus a proper multiple logistic regression model could not be performed. Our comparison of the patients who received concurrent RT+ICI vs. all those who did not means that the group that did not receive concurrent therapy was fairly heterogeneous. This would be one of the issues that multiple logistic regressions in a larger cohort could potentially address, but given the limitations of a retrospective review, we feel as though this was the best approach we could take with the current data that is available to us. A larger prospective cohort study is planned to further investigate the potential cardioprotective effect of thoracic RT given during ICI.

## Conclusion

This retrospective review does not show an increase in cardiac events with thoracic radiation given concurrently with ICI. In our limited study, as compared to those who received non-concurrent RT, patients with concurrent RT and ICI had a significantly lower rate of cardiac events in our cohort. This may suggest a potential cardioprotective effect of thoracic RT given during ICI, although this needs to be validated in a larger prospective study with a strong statistical design, and the underlying molecular mechanisms of this effect needs to be explored using basic models. If this possible cardioprotective effect is rigorously validated, then this might influence the findings of future clinical trials.

## Data availability statement

The raw data supporting the conclusions of this article will be made available by the authors, without undue reservation.

## Author contributions

CS wrote the manuscript and submitted the data. MM helped obtain and verify the cardiac toxicity data. AN and PW obtained and verified the immunotherapy data. AJ and MP obtained and verified the radiotherapy data and helped with the data analysis. All authors contributed to the article and approved the submitted version.
